# A critical review of analytical methods used for the chemical characterisation and quantification of phlorotannin compounds in brown seaweeds

**DOI:** 10.1002/pca.2851

**Published:** 2019-06-28

**Authors:** Lauren Ford, Katerina Theodoridou, Gary N. Sheldrake, Pamela J. Walsh

**Affiliations:** ^1^ School of Chemistry and Chemical Engineering Queen's University Belfast Belfast UK; ^2^ Institute of Global Food Security Queen's University Belfast Belfast UK

**Keywords:** polyphenols, seaweed, phlorotannins, analysis, algae

## Abstract

**Introduction:**

Phlorotannins, the phenolic compounds found in brown seaweeds, are a unique and diverse class of compounds showing a huge potential for food and pharmaceutical applications.

**Objective:**

This review will give an account of the colorimetric assays used and a discussion of their quantitative and qualitative analytical shortcomings. It will also discuss other more complex and modern analytical chemistry methods that are currently being developed to study phlorotannins. The purpose of this review is to increase awareness of these bioactive compounds and promote further development of robust analytical methods for use in biology, food science, pharmacology and biomedical and cosmeceutical sciences.

**Results:**

Whilst the biological activity and huge commercial potential of the phlorotannins has been widely reported throughout the literature, the chemical structures and reactivity of these compounds is still not well understood. The phlorotannin content of seaweed is usually characterised using colorimetric assays. However, although these methods give a reasonable overall estimation of the total phenolic content, they lack precision and specificity.

**Conclusion:**

This review highlights the strengths and weaknesses of commonly used colorimetric assays. Novel techniques are highlighted using more selective chemistry to identify this class of compounds.

## Abstract 2

Phlorotannins, the phenolic compounds found in brown seaweeds, are a unique and diverse class of compounds that have huge potential for food and pharmaceutical applications. This review will give an account of the analytical techniques used and a discussion of their quantitative and qualitative analytical shortcomings. The purpose of this review is to increase awareness of these bioactive compounds and promote further development of robust analytical methods for use in biology, food science, pharmacology and biomedical and cosmeceutical sciences.

## 1 INTRODUCTION

The growing human population across the globe has led to an increase in sourcing food and medicine in areas which have traditionally been overlooked. Macroalgae, known as seaweeds, are becoming an interesting crop as a sustainable alternative for many processes. Brown seaweeds in particular are of interest as they can be sustainably farmed.^1^, ^2^ The amount of seaweed produced on the island of Ireland is shown in Table 1. These marine plants contain a wide range of interesting natural products, such as bioactive phenolic compounds,^3^, ^4^ unsaturated fatty acids,^5^, ^6^, ^7^ alginate^8^, ^9^, ^10^ and biopolymers.^11^ Seaweed supplemented animal feeds offer nutritionally rich renewable feedstocks with several positive attributes to livestock health.^5^, ^10^, ^12^, ^13^, ^14^ These attributes include improved gut health and reduced faecal shedding.^13^, ^15^ It has also been reported in the literature that supplementing seaweed into the diet of pigs can decrease the need for antibiotics, which could slow or even avert the antibiotic resistance crisis the livestock industry is currently facing.^16^, ^17^ These antimicrobial and antibacterial effects have been reportedly due to the complex matrix of polyphenolic structures in the plant, collectively called phlorotannins.^18^, ^19^, ^20^ Brown seaweeds have also been shown to reduce methane emissions when fed to ruminants, which is of major importance in the context of the increasingly restricted emission targets that impinge on this area of intensive farming.^5^ Phlorotannins have been shown to be immensely valuable to the food and cosmetic industries due to their antioxidant and anti‐inflammatory properties.^21^, ^22^ Despite this, little is still understood about the characterisation and chemistry of these molecules. In order to gain a better understanding of the industrial value of phlorotannins, robust analytical methods are required to map the structure and size, of the compounds and their chemical linkages. Seaweed farming is of significant economic importance to immediate coastal areas^1^ and can generate higher revenue streams through the processing of high value products, such as phlorotannins.

**Table 1:** Commercial production of seaweed species in Ireland and expected annual seaweed harvest^70^

Seaweed species	Tonnes produced/year
*Ascophyllum nodosum*	25000
*Fucus serratus*	200
*Palmaria palmata*	<100
*Chondrus crispus/Mastocarpus stellatus*	<100
*Laminaria digitate*	<150
*Himanthalia elongata, Saccharina latissima, L. hyperborea, Ulva sp., Porphyra sp., Fucus vesiculosus, Alaria esculenta*.	<10

**Figure 1:**
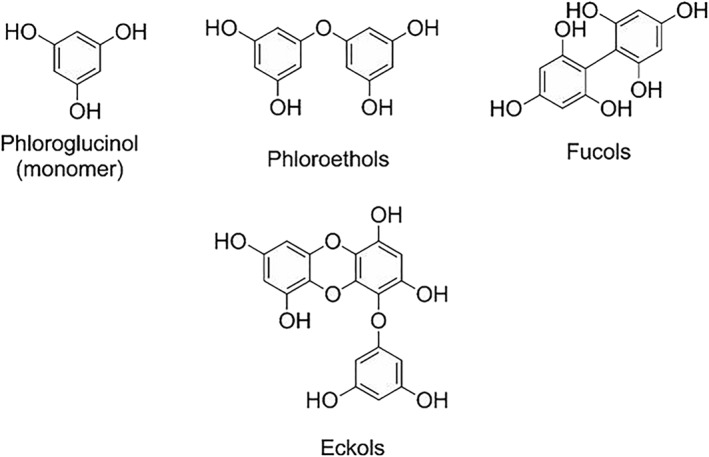
Phlorotannin structures

Phlorotannins display very similar characteristics to tannins produced by terrestrial plants but are structurally very different.^4^ Phlorotannins are polymeric structures of the monomer phloroglucinol (1,3,5‐hydroxybenzene) that are mainly linked through aryl‐aryl C‐C bonds or aryl‐ether C‐O bonds. The naming of phlorotannins is systematic based on the types of linkages between the aromatic groups, fucols only consist of aryl‐aryl linkages (Figure 1) whereas phloroethols are exclusively ether linkages through the phenolic oxygen.^23^ However, it is also possible to have both types of linkage in a single phlorotannin compound, which is named as a phlorofucol.^24^ Due to the variety of linkages and the array of molecular sizes of the phlorotannins, the number of possible structures is very high and as the molecular weight increases, the chemical complexity of the structure also increases. The molecular weight of this class of compounds can range from 126 to >10000 Da.^23^, ^25^ Phlorotannins have only been reported to be produced by brown seaweeds and are biosynthesised by the acetate malonate pathway.^26^, ^27^ They are very hydrophilic due to the presence of many phenolic OHs in their structure. This allows easy absorption of phlorotannins into biological systems when digested. Phlorotannins are most highly concentrated in the epidermal cortex of brown seaweed, however they are also found to be bound into the cell wall of the marine macroalgae.^27^ Phlorotannins are reported to contribute to the defence of the plant by acting as a herbivore deterrent, possibly by acting as an appetite suppressant.^28^ Phlorotannin concentrations vary from 0.5% to 20% of their dry weight, which can fluctuate with respect to season (e.g. changes in light exposure), environment (e.g. nutrient availability in the surrounding water) and also between species.^29^ It is noteworthy that there are some significant discrepancies between results in the literature and their methods of calculation. This is of particular concern from an analytical chemistry perspective, as yields quoted can mislead industry who are interested in commercialising particular phytochemicals.

Tannins from terrestrial plants are considered to have many health benefits including antioxidant properties, which have been claimed to help prevent degenerative diseases such as cancer and Alzheimer's.^19^ There have been many reviews addressing the biological activity of phlorotannins^21^, ^30^, ^31^, ^32^, ^33^ and their effects in food digestion for animals^12^ and humans has been a prevalent topic in recent literature. Phlorotannins have also been suggested to prevent the degradation of hyaluronic acid through the inhibition of HAase, which is linked to skin ageing, inflammation and also the migration of cancer cells.^26^ Antioxidants are sacrificial reducing agents, which minimise the effects of oxidation on a substrate, mechanism shown in Figure 3. Food rancidity is often caused by oxidation and hence the use of antioxidants can increase the shelf life of food. There are many diseases also associated with oxidative stress, and a diet high in antioxidants is thought to minimise the onset of these conditions.^28^ The oxidative stress in a biological system is caused by the presence of reactive oxygen species (ROS). These ROS occur naturally from the metabolism of cellular organisms or can be due to environmental factors such as smoke and pollution. If ROS are not quenched by the sacrificial reduction of an antioxidant they will react with carbohydrates, proteins, lipids or nucleic acids in biological systems and can disrupt their functionality.^28^ The phenolic content of marine (seaweed) and terrestrial plants is of interest across many fields studying plant matrices, due to their roles in anti‐oxidative reactions, astringency, bitterness, colour and browning reactions in food and formulation.^34^

## 2 TOTAL PHENOLIC CONTENT ASSAYS – QUANTITATIVE TECHNIQUES

Assays commonly used to study the polyphenolic content in terrestrial plants have also been extensively applied to study seaweeds.^8^, ^35^, ^36^, ^37^, ^38^ There are a number of assays which have been used to assess the content and activity of the polyphenolic phlorotannin compounds in seaweeds. The most commonly used colorimetric assays for the phlorotannin content are Folin–Ciocalteu (F‐C) and 2,4‐dimethoxybenzaldehyde (DMBA) assays (Table 2). These two assays vary slightly depending on both the way they interact with the target compounds and their sensitivity.

**Table 2:** Examples of the different extraction conditions and assays used to analyse the phlorotannin contents of seaweed species [equivalents: caffeic acid equivalents (CAE), gallic acid equivalents (GAE), phloroglucinol equivalents (PGE)]

Assay type	Species analysed	Time of year collected	Location collected	Storage/pre‐treatment	Extraction method	Equivalents expressed as	Reference
Folin–Ciocalteu	*Ascophyllum nodosum* and *Fucus vesiculosus*	Not specified	Not specified	Not specified	Commercially available extract. Typically prepared by hot water extraction and a series of filtrations.	CAE	71
Folin–Ciocalteu	*Laminaria digitata* , *Fucus serratus*, *Gracilaria gracilis* and *Codium fragile*	Spring 2011	County Clare, Republic of Ireland.	Stored at −18 °C	Solid–liquid extraction using four different solvent systems, namely cold water, hot water, ethanol/water (80:20) and methanol/water (70:30)	GAE	72
Folin–Ciocalteu	*Fucus vesiculosus*	Not specified	Archipelago Sea, southwest Finland.	Stored at −18 °C	Acetone/water (7:3) with ascorbic acid (0.3%) at room temperature with hexane pre‐treatment.	PGE	3, 34
Folin–Ciocalteu	*Ascophyllum nodosum*	March 2011	Supplied by “the Hebridean seaweed company”, Isle of Lewis, Scotland.	Not specified.	Ethanol/water (6:4).	PGE	68
Folin–Ciocalteu	*Fucus vesiculosus*	June 2010 and May 2012	Kiel Fjord, western Baltic Sea, Germany,	Stored at −60 °C	Acetone extraction on wet seaweed samples (seaweeds will contain a high amount of water).	PGE	47
Folin–Ciocalteu	*Sargassum fusiforme*	Not specified	Not specified	Not specified	Ethanol/water (1:1) extract. Different solid/liquid ratios studied and extraction times.	PGE	64
Folin–Ciocalteu	*Macrocystis pyrifera*	November 2011, March 2013 and June 2013	Puerto Montt, Chile.	Not specified	A range of solvents were compared methanol, ethanol, water, water/methanol 50:50, hexane/ethanol 88:12, ethanol/water 25:75 or 80:20, ethyl acetate/water 50:50, water/acetone 20:80 or 30:70 and methanol/chloroform 66:33% *v*/*v*. 55°C best extraction temperature.	GAE	46
Folin–Ciocalteu	*Fucus spiralis*	Collected between April and July 2014	Marques Neves beach, Peniche, Portugal.	Freeze dried samples after soaking in seawater.	Methanol extraction with a liquid–liquid partition with hexane to remove fats.	PGE	28
Folin–Ciocalteu	*Ascophyllum nodosum, Fucus spiralis, Fucus vesiculosus, Saccharina longicruris* and *Pelvetia canaliculata*	October 2008, August 2008	Nova Scotia, Canada. Except *Pelvetia canaliculata* which was harvested in Spiddal, Galway Bay, Ireland.	Freeze dried and ground upon harvest.	Methanol/water (8:2) defatted with dichloromethane.	PGE	23
DMBA and Folin–Ciocalteu	*Laminaria digitata*	Purchased in June 2015	Purchased from Bristol Botanicals, harvested in Scotland 2015.	Immediately air dried after harvest	Methanol/water (8:2) extraction at room temperature.	PGE	8
DMBA and Folin–Ciocalteu	*Fucus serratus* and *Ascophyllum nodosum*	February 2003	Corniche Armorique, St Efflam, France.	Stored at −20°C	Ethanol extraction on frozen wet seaweed samples. Temperature maintained at 0°C for the duration of extraction.	PGE	25

**Figure 2:**
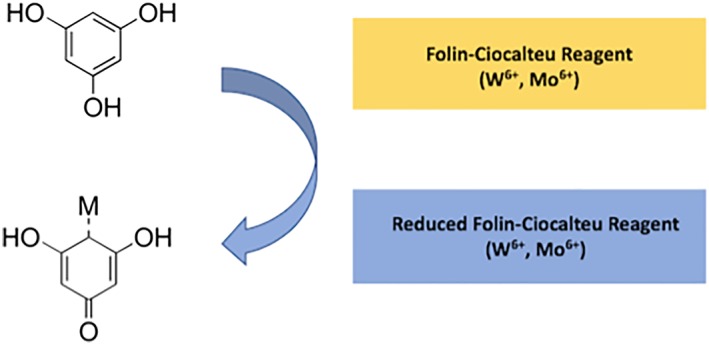
Diagram showing the reduction of the Folin–Ciocalteu reagent caused by the oxidation of the phenolics in a sample [Colour figure can be viewed at http://wileyonlinelibrary.com]

The F‐C assay is the most common assay used to quantify the phenolic content in both terrestrial plants and seaweeds.^8^, ^35^, ^38^, ^39^, ^40^, ^41^ The F‐C reagent is made up of a mixture of tungsten and molybdate.^42^ The F‐C assay relies on the transfer of electrons from phenolic compounds to phosphomolybdic/phosphotungstic acid complexes in alkaline conditions (Figure 2).^43^ The transfer of these electrons facilitates a colour change, which can then be detected at 760 nm in the visible spectrum.^44^ The blue colour which occurs upon reduction of the F‐C reagent is thought to be due to a coordinated molybdenum(V) species, although full characterisation of the structure is still unknown.^42^ Using the Beer–Lambert law the absorbance of the blue wavelength can then be calibrated using a standard compound. The phenolic content is calculated as equivalents of the standard.^43^ This method lacks sensitivity and its accuracy is questionable due to interference from other substrates (e.g. soluble sugars and proteins) in the plant matrix that can react with the F‐C reagents, causing artificially high values.^45^ Phenols, proteins, and thiols were all found to cause a reaction with the F‐C reagent in a study testing 80 different compounds.^42^ Many vitamins were also found to cause a response along with the inorganic ions Fe^2+^, Mn^2+^, I^−^, and SO^3+^.^42^ Therefore, the F‐C reaction is not sensitive to phenols but a measurement of anything in the matrix that is able to reduce, and therefore be oxidised itself. There is an assumption that the majority of reducing power in a plant matrix is due to phenolics and therefore the F‐C assay is used to give an approximation of the phenolic content.

DMBA reacts specifically with 1,3‐ and 1,3,5‐trihydroxybenzenes in a similar reaction to the vanillin assay to flavonoid compounds.^37^ This facilitates a colour change, which can be observed in the visible spectrum at 510 nm^37^ and quantified from a calibration curve of a standard. This is therefore more selective for the phenolic structures present in seaweed, as the specific functionality is required to react with the reagent, and so is less prone to overestimated content than the F‐C method. However, upon analysis of phlorotannins, this could possibly lead to an underestimation of their concentration as some phlorotannins are branched and can also have aryl linkages or fuhalols which contain additional hydroxyl groups in their structure in the 2, 4 or 6 positions. The mechanism of this reaction is not fully understood but DMBA is used for phlorotannin analysis over vanillin due to a stronger colour being produced. The reaction with the DMBA reagent must occur between the hydroxyl groups through the 2, 4 or 6 positions, therefore if these positions are substituted there will be no colour change and these phenolics will not be detected. This method must therefore be exercised with caution because some species could have more of these branches or linkages than others and therefore result in lower phenolic content reading than is actually the case. Steric hindrances could also cause lower reaction rates in larger phlorotannins with a larger degree of polymerisation.

Table 2 lists the various extraction methods and conditions used to measure the phenolic contents in seaweed. Table 2 highlights the variation between the protocols used in the literature, which then makes it difficult to directly compare between studies. Pre‐treatment of seaweed prior to extraction can also make a large difference on the results, and hence standardisation of the pre‐treatments when analysing phenolic content is also needed. Table 2 shows some studies use hexane as a pre‐treatment to remove lipophilic substances prior to F‐C analysis. This could help to remove fatty acids and other substances such as pigments that interfere with colorimetric assays. However, current protocols are not standardised, thus being another potential cause of discrepancies in results. Dichloromethane is also occasionally used to remove lipids from seaweeds; however, this process involves greater risk as dichloromethane is much more polar (solvent polarity index, 3.1) than hexane (solvent polarity index, 0.1) and hence could remove some compounds of interest.

There are a number of different parameters which need to be considered when analysing the phenolic content in the seaweeds, from collection through to measurement. Some studies fail to note the collection date, processing time, method and conditions of storage of seaweeds prior to analysis, all of which may influence the phenolic content. A study by Kirke *et al.,* reported that low molecular weight phlorotannins isolated from *Fucus vesiculosus* were stable up to 8 weeks in aqueous solutions at temperatures below 50°C.^24^ Prior to analysis, these samples were cleaned of epiphytes and other impurities, transported in cool conditions, frozen to −20°C, freeze‐dried, ground and stored in vacuum‐packed packages at −20°C. It is unclear from the literature, if phlorotannins are stable under other preparatory methodologies, as few studies report sample preparation prior to storage for analysis. In terms of the extraction, there are a number of parameters that can influence the extraction efficiency, which include, polarity of solvent, ratio of solvents, temperature of extraction, particle size of seaweed powders used in extraction and condition of samples (i.e. wet or dry samples). Leyton *et al*. compared a number of experimental extraction conditions and found acetone/water (70:30) to be the best extraction method for highest total phenolic content (TPC).^46^ This paper also assessed the optimum temperature for drying, extraction and pre‐treatment. They suggest the storage of dried seaweed at 4°C prior to use, a pre‐treatment with hexane (solid/liquid ratio of 5:1, *w*/*v*) and an extraction temperature of 55°C. Leyton *et al*. compared many parameters in their study however only looked at one species; *Macrocystis pyrifera*, and hence these conditions are optimised for this species. This paper highlights how different solvent systems, extraction temperatures and particle size affect the phenolic content, thus unless exactly the same extraction protocol is used, results from different publications are not comparable. Another consideration is the seaweed species, as different species produce phlorotannins with different linkages, functional groups, reactivity and sizes as previously mentioned. This may mean that the wide variability in structures may have different solubilities based on the structures of the polyphenols, and different polyphenols may be present in different species. Therefore, some species of seaweed may contain higher contents of smaller, less polar polyphenolic phlorotannins which would be more soluble in less polar solvents, whereas other species may contain larger, polar structures which would require more polar extraction solvents. Reactivity of the phlorotannins could also be very different from species to species and hence the temperature of extraction for one species may be optimum in terms of phenolic extraction and antioxidant activity but could cause degradation in another species leading to lower levels detected.

Another major difference in the extraction conditions are the temperature and time of extraction. The majority of extraction conditions involve room temperature conditions and overnight extractions.^32^, ^38^, ^47^ However, these conditions could vary from laboratory to laboratory, based on the room temperature in that part of the world. It is also known that the phlorotannins in brown seaweeds readily undergo oxidation and hence overnight extraction could lead to depleted results.^48^, ^49^, ^50^, ^51^

It is impossible to get a direct measurement of the total percentage weight of the phenolics, using these colorimetric assays. This is due to the vast quantities of different structures, hence a calibration curve of a known phenolic is created and used to express the phenolic quantities as an equivalent. There is a need to express the phenolic content with the same phenolic equivalent, so the results can be fit for comparison. The majority of the literature measuring the phenolic content of seaweed is currently using phloroglucinol (Table 2), which is logical as this is the monomer unit of the phlorotannin polymer being measured and hence would be most likely to show similar results. Phloroglucinol is the most widely used equivalent method to use when comparing between seaweed species, however, the majority of studies analysing terrestrial plants use gallic acid equivalents. Hence, if a comparison of the phenolic content of seaweed to terrestrial plants is required, a calibration of both phloroglucinol and gallic acid could be undertaken in order to get gallic acid equivalents and phloroglucinol equivalents.

Koivikko *et al*.^38^ studied different methods in their extraction procedure to combat the rapid oxidation of the phlorotannins. In this study, a range of conditions were tested, such as, adding ascorbic acid (an antioxidant), lowering the pH, and reducing the temperature. The addition of ascorbic acid is used because of the ease of oxidation of the phlorotannins, which could be protected by the antioxidant nature of ascorbic acid,^52^ it is thought there will be sacrificial oxidation to inhibit the oxidation of the phorotannins before analysis. Samples were then analysed by the F‐C assay and both normal and reversed phase high‐performance liquid chromatography (HPLC). Whilst the addition of ascorbic acid seemed beneficial when analysing by HPLC (more peaks observed due to less oxidation), it can cause interferences when using colorimetric assays, e.g. F‐C assay, as it would react with the F‐C reagent. Hence in order to avoid getting a more concentrated reading from the addition of ascorbic acid in the extraction, a reading of ascorbic acid only (at the same concentration that was added to the extraction) with the F‐C dye needs to be taken from the final calculation.

Table 3 exhibits the difference in total phenolic compounds reported in the literature for the same species of seaweeds when analysed using the F‐C assay. Due to the number of processing and extraction variables, it is impossible to conclude whether the differences in the phenolic contents are a result of these or due to seasonal or locational differences. Seasonality is an issue in these studies as it is well known that seaweeds produce different TPC values dependent on the time of year.^7^, ^36^, ^53^ The way the phenolic content is expressed can also cause issues because many different units are recorded in the literature, which also makes phenolic content difficult to compare between studies. For example, Table 2 shows phenolic content of seaweeds expressed in caffeic acid, gallic acid and phloroglucinol equivalents (CAE, GAE, PGE respectively), these can all then be expressed as % content, mg/g, PGE/100 g. Given these discrepancies it is difficult to make direct comparisons of studies in the literature.

**Table 3:** Seaweed species and the discrepancies in the literature in the total phenolic content (TPC) and the units of expression (PGE: phloroglucinol equivalents)

Seaweed species	Collection date and location	Extraction method	TPC and units expressed	Reference
*Ascophyllum nodosum*	March 2007, southwest Iceland.	7:3 (*v*/*v*) acetone/water extraction.	15.9 g PGE/100 g extract	73
*Ascophyllum nodosum*	Supplied by Portomuinos company, collected in August (no year given).	Dried in oven at 40°C, water extraction for analysis	0.96 g PGE/100 g extract	74
*Ascophyllum nodosum*	Nova Scotia, Canada between 2000 and 2003.	Methanol/water extract (1:1).	5.26% phenolic content	75
*Fucus serratus*	March 2007, southwest Iceland.	7:3 (*v*/*v*) acetone/water extraction.	24.0 g PGE/100 g extract	73
*Fucus vesiculosus*	March 2007, southwest Iceland.	7:3 (*v*/*v*) acetone/water extraction	24.2 g PGE/100 g extract	73
*Fucus vesiculosus*	Supplied by Portomuinos company, collected in August (no year given).	Dried in oven at 40°C, water extraction for analysis	1.15 g PGE/100 g extract	74
*Fucus vesiculosus*	Nova Scotia, Canada between 2000 and 2003.	Methanol/water extract (1:1).	23.21% phenolic content	75

## 3 PHLOROTANNIN QUANTIFICATION BY NMR SPECTROSCOPY – QUANTITATIVE TECHNIQUES

Proton nuclear magnetic resonance (^1^H‐NMR) spectroscopy can be used to quantify the TPC in a sample by using an internal standard. This has been carried out by Parys *et al*., who reported that NMR spectroscopy recorded higher TPC levels than the F‐C assay.^36^ However, both methods did follow the same trend in seasonal variations. The internal standard used was trimesic acid in 0.8 mL deuterated methanol and 0.2 mL deuterium oxide. A calibration curve was produced using phloroglucinol but it is important to note that when using NMR spectroscopy, the direct molar concentration of phlorotannin phenols are measured and it is not a calculated phloroglucinol equivalent. Since trimesic acid is used as the internal standard and not phloroglucinol, a comparison cannot be accurately made between this method and the other F‐C assays.

High‐resolution magic angle spinning (HR‐MAS) has also be used to quantitatively measure the phlorotannins in the brown algae; *Cystoseira tamariscifolia*.^54^ In this study solid‐state NMR was used to observe the presence of phloroglucinol in a solid sample of seaweed. The phloroglucinol singlet at 6.02 ppm was used to indicate presence within a sample with a relative intensity of three aromatic protons. This methodology was compared to ^1^H quantitative‐NMR (qNMR) using sodium trimethylsilylpropionate‐*d*_4_ (TSP) as an internal standard with an extraction of methanol/water (1:1, *v*/*v*). When compared to the F‐C assay, NMR showed consistently lower concentrations of phloroglucinol. This is expected, as explained previously, the F‐C assay is a relatively crude assay based on redox potential, it will also react with other phlorotannins as herein the concentration of only phloroglucinol is measured by NMR. The use of solid‐state NMR in the analysis of seaweeds is highly advantageous due to the fact that the seaweed can be analysed directly without the need for extraction with solvent. It therefore removes any matrix effects or extraction inefficiencies in analysis, however full quantification has not yet been achieved due to the lack of ability to use an internal standard in HR‐MAS, hence only the presence of phloroglucinol is detected rather than the concentration.^54^

Another study by the same authors also compared liquid chromatography electrospray ionisation tandem mass spectrometry (LC/ESI‐MS^*n*^) to ^1^H HR‐MAS in *Cystoseria,* a brown macroalage from the Fucaceae family.^55^ Whereas earlier ^1^H HR‐MAS in combination with ^1^H qNMR was used to qualitatively measure the concentration of phloroglucinol, the authors have suggested the use of this technique to be used qualitatively for use in taxonomy purposes. Fingerprints of the *Cystoseria* species were found to be useful in discriminating between the species tested, with the acceptation of two species; *C. foeniculacea* and *C. humilis* that could never be distinguished due to similar metabolic profiling.^55^

## 4 LIQUID CHROMATOGRAPHY OF PHLOROTANNINS AND HYPHENATED TECHNIQUES FOR QUANTITATIVE ANALYSIS

The use of liquid chromatography (LC) to quantify the phlorotannin compositions in macroalgal extracts is limited by the lack of commercially available standards. The only standard for calibration that is commercially available is the monomer phloroglucinol.

*Koivikko et al*. measured the concentration of phlorotannins through integration of peaks in a crude extract of *Fucus vesiculosus*.^38^ The F‐C assay was used to calculate the TPC of these compounds and then a Pearson correlation coefficient was calculated between the individual traces of the chromatogram and the contents of total phlorotannins. Further statistical analysis was performed to assess how well the variation in the phlorotannin chromatography profile can explain the variation of the content of total phlorotannins by conducting a multiple regression analysis. This attempt at quantification is however not complete as the area of the peak in the chromatographic profile is affected by the sensitivity of the compound to detection and the stability of the compounds under analytical conditions.^38^ Therefore further research needs to be performed with characterised standards in order to fully develop qualitative methods of high‐performance liquid chromatography (HPLC) analysis.

## 5 IDENTIFICATION OF PHLOROTANNIN STRUCTURE BY NMR SPECTROSCOPY – QUALITATIVE ANALYSIS

NMR spectroscopy is a good method for identifying purified phlorotannins. The structures of the phlorotannins are very similar, and hence the different compounds have only subtle differences in chemical shift, making them difficult to differentiate. Glombitza and coworkers have successfully identified numerous purified phlorotannin structures by NMR spectroscopy.^48^, ^49^, ^50^ Due to the complexity of the spectra associated with these compounds, it is only possible to structurally identify the smaller polyphenolic structures of three to eight aromatic rings by NMR spectroscopy. A pre‐treatment step can be used that involves the precipitation of the larger phlorotannins with ether and petrol, so isolating smaller compounds for analysis.^56^ Due to the instability of the phlorotannins, and to make the NMR analysis easier, the compounds are usually acetylated using acetic anhydride and pyridine.^25^, ^48^, ^49^, ^50^, ^51^, ^57^ The acetylation of the hydroxyls of the phenolics prevents the keto‐enol tautomerisation and thereby suppresses oxidation. In these types of phenolic compounds, the hydrogens between two phenolic groups on an aromatic ring are susceptible to hydrogen‐deuterium exchange with solvents such as deuterium oxide and deuterated methanol, which can result in diminished or fully exchanged peaks in the spectra. However, acetylation of the phlorotannins prevents this exchange from occurring, also because the keto‐enol tautomerisation is prevented. Before compounds can be identified by NMR spectroscopy they must first be purified by chromatography. Acetylation of the phlorotannins changes the polarity of the compounds and hence, once acetylated, the phorotannins can then be separated by normal phase silica chromatography using solvent systems of *n‐*hexane, chloroform, methanol or ethanol.^57^ Thin layer chromatography, can be used to measure the retention factor (Rf) values of the compounds with a vanillin and sulphuric acid stain for visualisation.^58^ Preparative thin layer chromatography can also be used to separate compounds of small samples.

^1^H‐NMR spectroscopy is a powerful tool used for determining the structures of the phenolic compounds present in the seaweed species being analysed. The aromatic protons resonate between 6.0 and 7.5 ppm. The acetyl methyl groups will show as sharp singlet peaks between 2 and 3 ppm, with integration of these signals being a useful tool to establish the number of free hydroxyl groups in the original unprotected phenolics. Table 4 displays the different ring systems that can occur in phenolic compounds. The pattern of the coupling constant and integration of peaks can be used to identify ring structures in unknown phenolics.

**Table 4:** The ring systems used to describe the aromatic rings in phlorotannin structures

Ring system	Ring structure
AB_2_ – One hydrogen in the A position, two hydrogens in the B position	(1) 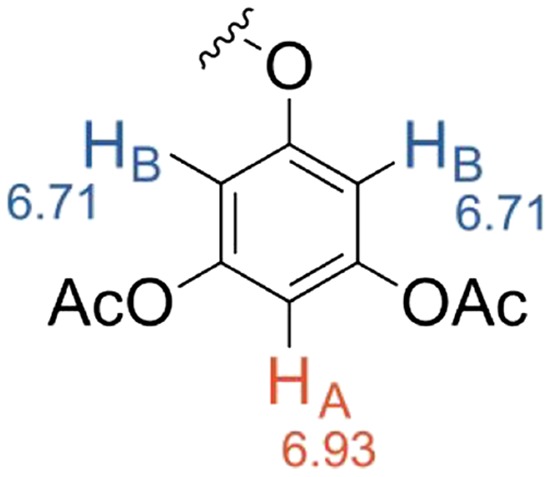
AB – Two hydrogens in different environments	(2) 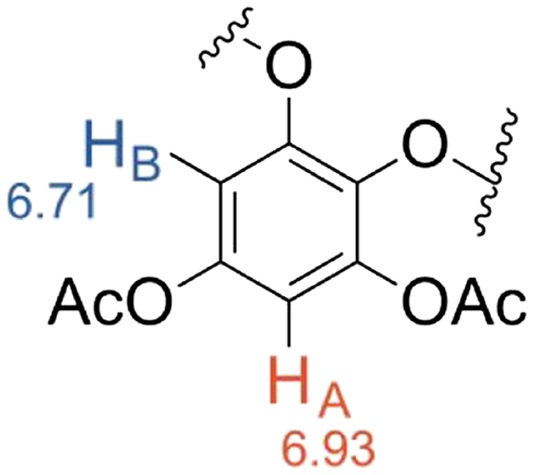

Whilst ^1^H‐NMR spectroscopy is useful for individual highly purified compounds or for quantification, it is less useful for the identification of individual minor phlorotannin components in complex mixtures. Heteronuclear multiple bond correlation can be utilised with ^13^C‐NMR spectroscopy to give more clarity on these structures by linking the aromatic hydrogen signals to the carbon signals. Characteristic ^13^C‐NMR signals of phlorotannins are displayed in Table 5. This heteronuclear correlation NMR spectroscopic method is useful for identifying the class of phlorotannin from a sample matrix (monomer, fucol, phloroethol, fulahol, fucophloroethol, etc.).^,80^

**Table 5:** ^13^C signals useful for identification of phlorotannin structural identification^76^

^13^C‐NMR chemical shift (ppm)	Functional group
100	Aryl‐aryl signals
120–150	Signals for ether linkages and hence indicates the presence of phloroethols type phlorotannins,
145–155	Signals for hydroxyl groups additional to the 1,3,5‐hydroxyl groups originating from phloroglucinol which indicates the presence of fuhalols

**Figure 3:**
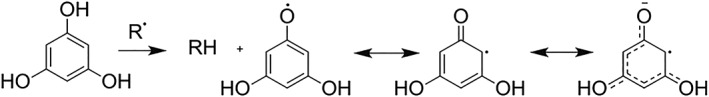
Reaction of polyphenolic ring with a reactive radical species

Heteronuclear single quantum coherence spectroscopy (HSQC) and heteronuclear multiple bond correlation spectroscopy (HMBC) two‐dimensional (2D) NMR spectroscopic techniques have been used by Cérantola *et al*. to display the presence of fucol and fucophloroethol structures in extracts of *Fucus spiralis*.^59^ This method has also been used to determine the ratio of ether linkages compared to aryl linkages of a sample extracted from *Laminaria digitata*.^8^ This methodology is very important as the structural integrity of these compounds could have large effects on their biological activity Figure 3.

**Figure 4:**
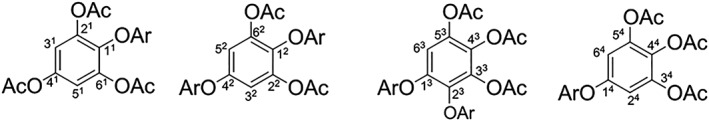
Structures of phlorotannin aromatic rings numbered as previously recorded by Glombitza et al.^18^

The ^13^C‐NMR chemical shifts from the fuhalols, which have been identified in *Bifurcaria bifurcata*, are shown in Table 6. The different monomers, from which the larger phlorotannin structures are constructed, are displayed in Figure 4 and their carbons numbered; this is not in accordance to IUPAC numbering but a system that has been used previously by Glombitza ad coworkers.^60^

**Table 6:** ^13^C‐NMR chemical shifts of fuhalols from *Bifurcaria bifurcata*

^13^C‐NMR chemical shift (ppm)	Assignment based on numbers in Figure 5
168–166	Carbonyl (acetyl groups)
154.7	1^4^
153.8–154.4	4^2^
147.9–148.0	1^3^
146.8	4^1^
144.0	3^4^, 5^4^
143.8	2^1^, 6^1^, 2^2^, 6^2^
140.2–140.3	5^3^
138.0	3^3^
136.6	1^1^
134.8	2^3^
134.3–134.6	1^2^
130.9–131.5	4^3^
115.1	3^1^, 5^1^
109.5–109.8	3^2^, 5^2^
109.1	6^3^, 2^3^, 6^4^, 2^4^
21–20	Methyls (acetyl groups)

Note: Ether linkages for phenol linkages *meta* and *ortho* to an acetyl group are highlighted in green.

**Figure 5:**
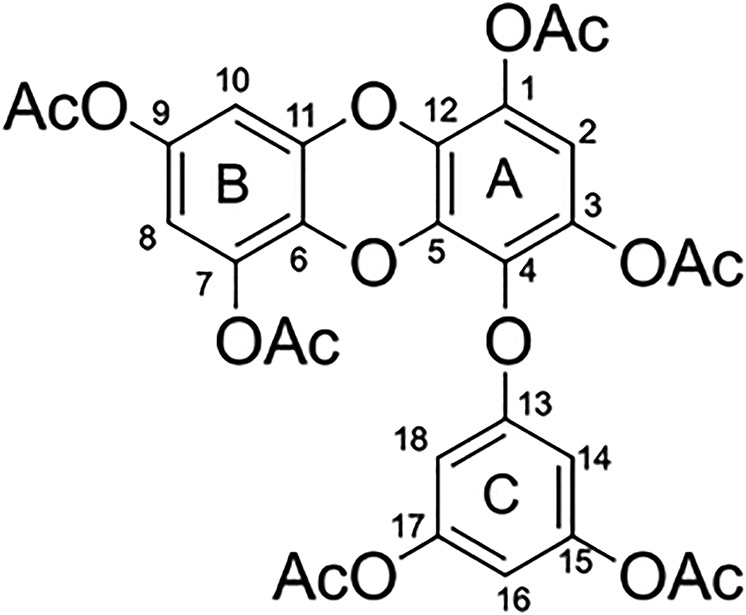
Eckol numbering for ^13^C NMR spectra assignment^18^

From Table 6 it is observed that the ether linkages correspond to peaks in the region 147.9–154.7 ppm for phenol linkages *meta* to an acetyl group and 136–134 ppm for phenol linkages *ortho* to an acetyl group. The carbon NMR data can also be seen for the Eckol structure shown in Figure 5 the values of which are depicted in Table 7. Here it is shown that the eckol type linkages are between 130–145 ppm on a ^13^C NMR spectra. From Table 7 it is observed that the eckol linkages are very close in chemical shift to the acetylated phenolics, hence 2D NMR is needed to distinguish them.

**Table 7:** ^13^C‐NMR spectral data for the assignment of an eckol structure

Chemical shift (ppm)	Assignment based on numbers in Figure 6.
166–168	Carbonyls from acetyl groups
157.9	13
151.7	15, 17
145.9	9
141.7	11
139.2	7
138.7	3
136.4	5
134.9	1
133.0	4
131.9	6, 12
112.7	2, 8
110.2	16
108.0	10
106.3	14, 18

Note: dibenzodioxin ether carbon signals are highlighted in blue.

^13^C‐NMR spectral data for eckol structures showing that the dibenzodioxin ether carbon signals are within the range 131–141 ppm (highlighted). The data shown in Tables 7 and 4 shows the importance of using both ^1^H‐ and ^13^C‐NMR spectra to identify phlorotannin structures, either purified or in crude extracts. Using the intensity of the signals it is possible to identify the ratio of linkages and chemical shifts corresponding to certain chemical groups which can be used to determine the abundance of the different types of phlorotannin in a mixture. However, achieving good quality ^13^C‐NMR spectral data of a complex mixture can be difficult.

## 6 MASS SPECTROMETRY IN QUALITATIVE ANALYSIS AND IDENTIFICATION OF PHLOROTANNINS – QUALITATIVE ANALYSIS

Mass spectrometry (MS) is a powerful tool in the analysis and identification of phlorotannin compounds. It is mostly regarded as a qualitative tool due to the lack of standards available to counteract quantitative hurdles in MS such as matrix effects. Tandem mass spectrometry or MS/MS has been frequently used coupled to various LC systems to observe the fragmentation pattern of the compounds as they elute on the column.^23^, ^61^, ^62^ Assignment of phlorotannins is usually based on the size of the parent ion for example; typical [M‐H]^−^ fragmentation of a phlorotannin of five phloroglucinol units (PGU) will be *m/z* 621 parent ion. With typical fragments of *m/z* 603 corresponding to the loss of water, *m/z* 495 corresponding to the loss of phloroglucinol; and then the loss of further phloroglucinol units *m/z* 373.^63^

The nature of the ionisation source is also important for the analysis of phlorotannin compounds from seaweed extracts. Electrospray ionisation (ESI) is most commonly used for LC‐MS techniques and is a very good soft ionisation technique; meaning that it enables the compounds to be detected without too much fragmentation (unless using MS/MS).^8^, ^46^, ^64^ Larger compounds of up to 27 degrees of polymerisation (DP) can be ionised and analysed using matrix assisted laser desorption/ionisation‐time‐of‐flight (MALDI‐ToF).^8^ MALDI is an ionisation technique that uses laser energy to produce ions from large molecules without fragmentation, hence is required for phlorotannins with higher DP values. High‐resolution mass spectrometry (HRMS) can also measure larger phlorotannins of up to 49 DP due to the peaks appearing as multiple charged ions as the number of free hydroxyl groups increases. In this study, a large phlorotannin with 34 PGU was identified, by a *m*/*z* ratio of 1405.85, however the pattern of the +1 ^13^C isotope peak shows an *m*/*z* ratio of 0.33 which indicates a triple charge corresponding to a molecular weight of 4218.56 g/mol. Without HRMS the isotope pattern could not have been deciphered and hence the chemical formula or molecular weight could not have been obtained.^8^

Both ESI and MALDI use a matrix in the ionisation of compounds for analysis. Direct analysis of phlorotannins in MS techniques can sometimes lead to very weak signals due to contamination of other classes of compounds in the extracts; for example, polysaccharides. Different techniques have been employed to overcome these issues by purifying the phlorotannins. Samples have been previously purified by molecular weight cutoff dialysis^65^ and solid‐phase extraction.^35^ The disadvantage of these techniques is that some phlorotannin compounds could be separated from the bulk due to the widely varying size and structures of this class of compounds; and hence would not undergo analysis.

## 7 LIQUID CHROMATOGRAPHY OF PHLOROTANNINS AND HYPHENATED TECHNIQUES FOR QUALITATIVE ANALYSIS

The large number of very similar compounds in a typical extract makes analysis and identification of the individual structures very difficult. The similarities of the different phlorotannin structures also poses problems for separation of the mixtures by conventional methods such as column chromatography. This makes separation of individual phlorotannins very difficult by HPLC but, not impossible. Reversed phase chromatography can be a useful tool in separating very structurally similar compounds in complex mixtures however, due to the polar nature of polyphenolic phlorotannin compounds they elute very fast. Normal phase chromatography involves the use of a polar stationary phase but use of this chromatography method can lead to the polar phlorotannins being difficult to elute at all due to high interaction with the stationary phase.

One study has shown separation with a hydrophilic interaction liquid chromatography (HILIC) column.^23^ HILIC columns utilise a hydrophilic stationary phase with reversed‐phase type eluents and hence can be described as a variant of normal phase HPLC which overlaps with reversed phase HPLC. HILIC columns are therefore ideal at separating out phlorotannins because they will have strong interaction with the polar stationary phase but can be eluted with reversed‐phase type eluents. This study showed a big difference in the phlorotannin composition between the species *Fucus vesiculosus*, *Fucus spiralis*, *Ascophyllum nodosum* and *Saccharina longicruris*. *Fucus vesiculosus* displayed the highest concentration of low molecular weight phlorotannins (< 1200 Da).^23^ In this study the chromatograph was coupled to a high‐resolution mass spectrometer in order to generate more structural information on the peaks.

More recently Ultra‐performance liquid chromatography mass spectrometry (UPLC‐MS) has been employed to determine the metabolite profile of the phlorotannins in three species of brown macroalgae. UPLC has been developed to endure higher system back pressures than conventional HPLC and thus much smaller column particle sizes can be used, which enhances speed and sensitivity of analysis.^66^ *Tierney et al*. used UPLC‐MS to evaluate the polyphenolic profile of *Ascophyllum nodosum*, *Pelvetia canaliculata* and *Fucus spiralis*.^65^ This work involved extraction with cold water (CW) and ethanolic water (EW) (8:2) and the phlorotannins were concentrated using molecular weight cutoff dialysis and reversed phase flash chromatography. The isolation of pure phlorotannin extracts is important for the characterisation of metabolites by UPLC‐MS as weak total ion current signals are observed upon contamination from polar polysaccharides.^65^ UPLC‐MS in this case also allows analysis of the DP to be calculated for identified phlorotannins. This study found that the predominant peaks in *Ascophyllum nodosum* are visible at *m/z* 745 (DP of 6), 1117 (DP of 9), 1241 (DP of 10), 1365 (DP of 11) and 1490 (DP of 12). This differed drastically to the profile of *Fucus spiralis* in which phlorotannin polymers had the most distinguishable peaks of *m/z* 497 (DP of 4), 621 (DP of 5) and 745 (DP of 6.^65^

**Figure 6:**
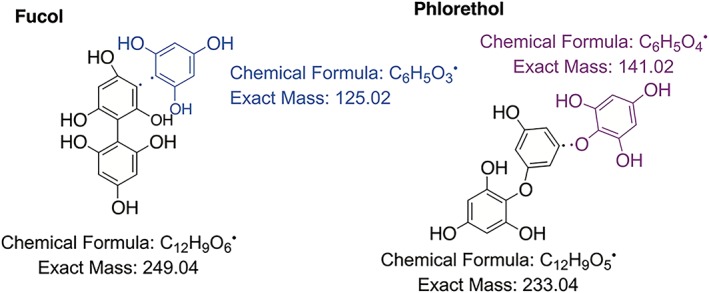
Characteristic fragment patterns of phlorotannins observed by mass spectrometry [Colour figure can be viewed at http://wileyonlinelibrary.com]

Although HPLC cannot be used to fully identify the structure of the phlorotannin and the position of linkages, valuable information on the distribution, type and size of phlorotannins can be obtained.^8^ The fragmentation behaviour of the phlorotannins can give information on the linkage type based on the charged fragments observed in the mass spectrum.^2^, ^8^ Some characteristic fragments are displayed in Figure 6. Another study separated the small (< 2 kDa) phlorotannins from *Ascophyllum nodosum* on a C18 column into two distinct peaks using diode array detector (DAD).^67^ However, separation and identification of phlorotannins by HPLC fitted with a DAD is complicated even further by the lack of standards available for comparison.^68^ Whilst DAD detection can be useful for analysis of compounds with similar molecular weights but different electronic distribution in the chromophore, leading to different UV spectra, standards or literature data are needed for comparison if the compounds are to be identified. The composition of the mixtures in the peaks in this study were not conclusively identified due to lack of standards available. To add to this, the phlorotannin structures are very prone to oxidation during extraction due to the increased tendency of multiple phenolic groups on one aromatic ring to tautomerise to the more reactive keto form. The instability of the phlorotannins prior to analysis can affect the reproducibility of the results when performing these experiments.

Two‐dimensional LC techniques have been employed to assess the complex matrix of phlorotannin compounds in seaweed extracts from the brown seaweed *Cystoseria abies‐marina*.^63^ Phlorotannins were separated using this technique first by HILIC and then by reversed phase separation in the second dimension. The employment of this technique allowed for separation and identification of 50 compounds when analysing brown seaweed; *Cystoseria abies‐marina*. 2D‐LC used in this research was coupled to DAD and MS/MS to allow for identification and elucidation of the phlorotannin compounds detected. Optimisation of this technique resulted in the use of a microbore column with diol particles in the first dimension which allowed for a very low flow rate to be used in order to give enough time for the second dimension to be completed.^63^ Two different reversed phase columns were selected for analysis of the algal extracts in the second dimension; C18 column which have routinely been used previously,^4^, ^46^, ^67^ and a Kintex pentafluorophenyl (PFP) column which is more novel in this application. The different columns used in these experiments differ in polarity and electron density and therefore would be expected to show different separations in experimental conditions. In this setup phlorotannins are separated in terms of DP in the first dimension and then in terms of their relative hydrophobicity in the second dimension.^63^ C18 column used in the second dimension appeared to separate the phlorotannins more efficiently than the PFP column, this is due to the fluorine atoms being in the periphery of a phenyl ring being much more electronegative than the long C18 carbon chain.^63^ Phlorotannins of between 5 and 17 PGU were identified using this method and tentatively identified using MS and MS/MS spectra. A flavonoid compound was also identified in this matrix along with eight other unidentified compounds that did not match anything in the MS database. This 2D LC method has also been used to analyse phlorotannins from North Atlantic *Sargassum muticum* samples.^69^ In this study extracts from different locations were tested for their anti‐proliferative activity against human colon cancer cells, the samples with highest activity were analysed with 2D LC‐DAD‐MS‐MS/MS.^69^ This technique allows for profiling of the phlorotannin extracts, which cannot be done using traditional colorimetric analysis methods and allows observations of different phlorotannin compounds depending on where the seaweed is growing even though all samples were from the same species. This work suggests that the activity of the extract is due to the chemical structures of the phlorotannin compounds and 2D LC‐MS provides a useful method of qualitative analysis for these complex matrixes.^63^

In conclusion, robust analytical techniques to quantify and characterise polyphenols in seaweeds is vital to industries to translate seaweed bioactives commercially. Characterisation of the structures is tentatively carried out by HRMS coupled to HPLC and parent ion peaks compared to those found in the literature. However, if full elucidation of the linkage position and isomeric forms is desired NMR spectroscopy is currently the only effective method of analysis. ^13^C‐NMR spectral data can be used to calculate the ratio of linkages present in a seaweed matrix which can be done in a complex mixture and does not need purification beforehand. ^1^H‐NMR spectra can also be used to quantify the phlorotannin content in extracts rapidly and with reasonable accuracy by use of an internal standard. In the NMR spectroscopic method, only the peaks due to phlorotannins are measured whereas F‐C analysis, by comparison, relies on a secondary effect and can give positive results for other compounds in the extract. However, ^1^H‐NMR spectroscopy has only been demonstrated to be effective for *Ascophyllum nodosum* and *Cystoseria* species and seaweeds naturally lower in phlorotannins may be more difficult to analyse by this method. More species of seaweeds must be analysed using this method to test the reproducibility in other matrixes.

Colorimetric assays could be a high throughput, easy and cost‐effective tool for measuring phlorotannin content but robust work needs to be done in order to standardise a method to make results comparable between studies. Many papers have focused on optimising methods for one species of seaweed, but more species need to be used in these types of studies to ensure that optimised methods are suitable for most brown seaweed species.

**Figure 7:**
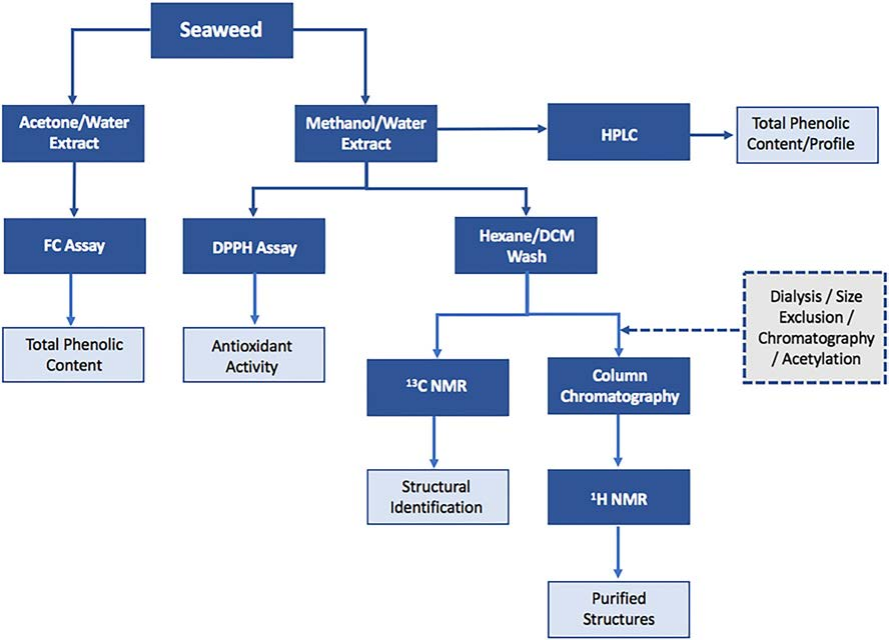
Schematic to show the sample preparation steps involved with each analysis technique [Colour figure can be viewed at http://wileyonlinelibrary.com]

HPLC, especially when coupled to MS, is a useful technique to study high and low molecular weight phlorotannins and their distributions in an extract. However, often specialised equipment is required to run techniques such as MALDI‐ToF and HRMS which are required to detect and identify larger phlorotannins. Figure 7 is a schematic showing the sample preparation step required for the analytical techniques covered in this review. From this schematic it can be seen that the assay analysis is by far the fastest and simplest method. However, this gives the least reliable results as there are too many variables which can have an effect on the data, and hence it is difficult to compare the results from different studies. HPLC would be a very good method to use if there were more standard compounds of phlorotannins available to generate reliable libraries for comparison. Most laboratories have access to HPLC‐DAD whereas MS detection might not be as widely available. A really useful step forward for the research community in this area would be a study to identify the common phlorotannin compounds by NMR spectroscopy and then link these structures to HPLC retention times and UV spectral data.

## References

[1] Wood D , Capuzzo E , Kirby D , Mooney‐McAuley K , Kerrison P . UK macroalgae aquaculture: what are the key environmental and licensing considerations? Mar Policy. 2017;83:29‐39.

[2] Kerrison PD , Stanley MS , Edwards MD , Black KD , Hughes AD . The cultivation of European kelp for bioenergy: site and species selection. Biomass Bioenergy. 2015;80:229‐242.

[3] Koivikko R , Loponen J , Pihlaja K , Jormalainen V . High‐performance liquid chromatographic analysis of phlorotannins from the brown alga *Fucus vesiculosus* . Phytochem Anal. 2007;18(4):326‐332. 17623367 10.1002/pca.986

[4] Isaza Martínez JH , Torres Castañeda HG . Preparation and chromatographic analysis of phlorotannins. J Chromatogr Sci. 2013;51(8):825‐838. 23592824 10.1093/chromsci/bmt045

[5] Belanche A , Jones E , Parveen I , Newbold CJ . A metagenomics approach to evaluate the impact of dietary supplementation with *Ascophyllum nodosum* or *Laminaria digitata* on rumen function in Rusitec fermenters. Front Microbiol. 2016;7(MAR):1‐14. 27014222 10.3389/fmicb.2016.00299 PMC4785176

[6] Susanto E , Fahmi AS , Abe M , Hosokawa M , Miyashita K . Lipids, fatty acids, and Fucoxanthin content from temperate and tropical brown seaweeds. Aquat Procedia. 2016;7:66‐75.

[7] Schmid M , Guihéneuf F , Stengel DB . Fatty acid contents and profiles of 16 macroalgae collected from the Irish coast at two seasons. J Appl Phycol. 2014;26(1):451‐463.

[8] Vissers AM , Caligiani A , Sforza S , Vincken JP , Gruppen H . Phlorotannin composition of *Laminaria digitata* . Phytochem Anal. 2017;28(6):487‐495. 28612431 10.1002/pca.2697

[9] Dusseault J , Tam SK , Ménard M , et al. Evaluation of alginate purification methods: effect on polyphenol, endotoxin, and protein contamination. J Biomed Mater Res ‐ Part a. 2006;76(2):243‐251. 10.1002/jbm.a.30541 16265647

[10] Makkar HPS , Tran G , Heuzé V , et al. Seaweeds for livestock diets: a review. Anim Feed Sci Technol. 2016;212:1‐17.

[11] Angell AR , Angell SF , de Nys R , Paul NA . Seaweed as a protein source for mono‐gastric livestock. Trends Food Sci Technol. 2016;54:74‐84.

[12] Evans FD , Critchley AT . Seaweeds for animal production use. J Appl Phycol. 2014;26(2):891‐899.

[13] Bach SJ , Wang Y , McAllister TA . Effect of feeding sun‐dried seaweed (*Ascophyllum nodosum*) on fecal shedding of *Escherichia coli* O157:H7 by feedlot cattle and on growth performance of lambs. Anim Feed Sci Technol. 2008;142(2):17‐32.

[14] Bleakley S , Hayes M . Algal proteins: extraction, application, and challenges concerning production. Foods. 2017;6(5):33‐67. 10.3390/foods6050033 PMC5447909 28445408

[15] Berge AC , Wierup M . Nutritional strategies to combat salmonella in mono‐gastric food animal production. Animal. 2012;6(4):557‐564. 22436270 10.1017/S1751731111002217

[16] Huang Q , Liu X , Zhao G , Hu T , Wang Y . Potential and challenges of tannins as an alternative to in‐feed antibiotics for farm animal production. Anim Nutr. 2018;4(2):137‐150. 30140753 10.1016/j.aninu.2017.09.004 PMC6104569

[17] Siva R , Palackan MG , Maimoon L , et al. Evaluation of antibacterial, antifungal, and antioxidant properties of some food dyes. Food Sci Biotechnol. 2011;20(1):7‐13.

[18] Glombitza KW , Vogels HP . Antibiotics from algae. XXXV. Phlorotannins from *Ecklonia maxima* . Planta Med. 1985;51(4):308‐312. 17340520 10.1055/s-2007-969498

[19] Lopes G , Sousa C , Silva LR , et al. Can phlorotannins purified extracts constitute a novel pharmacological alternative for microbial infections with associated inflammatory conditions? PLoS ONE. 2012;7(2):1‐9. 10.1371/journal.pone.0031145 PMC3271118 22319609

[20] Glombitza K‐W . Antibiotics from algae. Mar Algae Pharm Sci. 1979;28:303‐342.

[21] Zou Y , Qian Z‐J , Li Y , Kim M‐M , Lee S‐H , Kim S‐K . Antioxidant effects of phlorotannins isolated from *Ishige okamurae* in free radical mediated oxidative systems. J Agric Food Chem. 2008;56(16):7001‐7009. 18616277 10.1021/jf801133h

[22] Wijesinghe WAJP , Jeon YJ . Biological activities and potential cosmeceutical applications of bioactive components from brown seaweeds: a review. Phytochem Rev. 2011;10(3):431‐443.

[23] Steevensz AJ , MacKinnon SL , Hankinson R , et al. Profiling phlorotannins in brown macroalgae by liquid chromatography‐high resolution mass spectrometry. Phytochem Anal. 2012;23(5):547‐553. 22383068 10.1002/pca.2354

[24] Kirke DA , Smyth TJ , Rai DK , Kenny O , Stengel DB . The chemical and antioxidant stability of isolated low molecular weight phlorotannins. Food Chem. 2017;221:1104‐1112. 27979066 10.1016/j.foodchem.2016.11.050

[25] Parys S , Rosenbaum A , Kehraus S , Reher G , Glombitza KW , Konig G . Evaluation of quantitative methods for the determination of polyphenols in algal extracts. J Nat Prod. 2007;70(12):1865‐1870. 18052031 10.1021/np070302f

[26] Ferreres F , Lopes G , Gil‐Izquierdo A , et al. Phlorotannin extracts from fucales characterized by HPLC‐DAD‐ESI‐MS*n*: approaches to hyaluronidase inhibitory capacity and antioxidant properties. Mar Drugs. 2012;10(12):2766‐2781. 23222802 10.3390/md10122766 PMC3528125

[27] Arnold TM , Targett NM . Marine tannins: the importance of a mechanistic framework for predicting ecological roles. J Chem Ecol. 2002;28(10):1919‐1934. 12474891 10.1023/a:1020737609151

[28] Pinteus S , Silva J , Alves C , Horta A , Thomas OP , Pedrosa R . Antioxidant and cytoprotective activities of fucus spiralis seaweed on a human cell *in vitro* model. Int J Mol Sci. 2017;18(2):1‐14. 10.3390/ijms18020292 PMC5343828 28146076

[29] Caro Y , Anamale L , Fouillaud M , Laurent P , Petit T , Dufosse L . Natural hydroxyanthraquinoid pigments as potent food grade colorants: an overview. Nat Prod Bioprospecting. 2012;2(5):174‐193.

[30] Lopes G , Andrade PB , Valentão P . Phlorotannins: towards new pharmacological interventions for diabetes mellitus type 2. Molecules. 2017;22(1):1‐21. 10.3390/molecules22010056 PMC6155720 28042834

[31] Wang Y , Xu Z , Bach SJ , Mcallister TA . Effects of phlorotannins from *Ascophyllum nodosum* (brown seaweed) on *in vitro* ruminal digestion of mixed forage or barley grain. Anim Feed Sci Technol. 2008;145(1‐4):375‐395.

[32] Koivikko R , Loponen J , Honkanen T , Jormalainen V . Contents of soluble, cell‐wall‐bound and exuded phlorotannins in the brown alga *Fucus vesiculosus*, with implications on their ecological functions. J Chem Ecol. 2005;31(1):195‐212. 15839490 10.1007/s10886-005-0984-2

[33] Sugiura Y , Matsuda K , Yamada Y , et al. Isolation of a new anti‐allergic phlorotannin, phlorofucofuroeckol‐B, from an edible brown alga, *Eisenia arborea* . Biosci Biotechnol Biochem. 2006;70(11):2807‐2811. 17090915 10.1271/bbb.60417

[34] Singleton VL , Orthofer R , Lamuela‐Raventós R . Analysis of total phenols and other oxidation substrates and antioxidants by means of Folin–Ciocalteu reagent. Methods Enzymol. 1974;299:152‐178.

[35] Tanniou A , Vandanjon L , Incera M , et al. Assessment of the spatial variability of phenolic contents and associated bioactivities in the invasive alga *Sargassum muticum* sampled along its European range from Norway to Portugal. J Appl Phycol. 2014;26:1215‐1230.

[36] Parys S , Kehraus S , Pete R , Küpper FC , Glombitza KW , König GM . Seasonal variation of polyphenolics in *Ascophyllum nodosum* (Phaeophyceae). Eur J Phycol. 2009;44(3):331‐338.

[37] Stern JL , Hagerman AE , Steinberg PD , Winter FC , Estes JA . A new assay for quantifying brown algal phlorotannins and comparisons to previous methods. J Chem Ecol. 1996;22(7):1273‐1293. 24226084 10.1007/BF02266965

[38] Koivikko R , Eränen JK , Loponen J , Jormalainen V . Variation of phlorotannins among three populations of *Fucus vesiculosus* as revealed by HPLC and colorimetric quantification. J Chem Ecol. 2008;35:57‐64. 10.1007/s10886-007-9410-2 18157573

[39] Sánchez‐Rangel JC , Benavides J , Heredia JB , Cisneros‐Zevallos L , Jacobo‐Velázquez DA . The Folin–Ciocalteu assay revisited: improvement of its specificity for total phenolic content determination. Anal Methods. 2013;5(21):5990.

[40] Peschel W , Sánchez‐Rabaneda F , Diekmann W , et al. An industrial approach in the search of natural antioxidants from vegetable and fruit wastes. Food Chem. 2006;97(1):137‐150.

[41] Wootton‐Beard PC , Moran A , Ryan L . Stability of the total antioxidant capacity and total polyphenol content of 23 commercially available vegetable juices before and after *in vitro* digestion measured by FRAP, DPPH, ABTS and Folin–Ciocalteu methods. Food Res Int. 2011;44(1):217‐224.

[42] Everette JD , Bryant QM , Green AM , Abbey YA , Wangila GW , Walker RB . Thorough study of reactivity of various compound classes toward the Folin–Ciocalteu reagent. J Agric Food Chem. 2010;58(14):8139‐8144. 20583841 10.1021/jf1005935 PMC4075968

[43] Ainsworth EA , Gillespie KM . Estimation of total phenolic content and other oxidation substrates in plant tissues using Folin–Ciocalteu reagent. Nat Protoc. 2007;2(4):875‐877. 17446889 10.1038/nprot.2007.102

[44] Singleton VL , Orthofer R , Lamuela‐Raventós RM . Analysis of total phenols and other oxidation substrates and antioxidants by means of Folin–Ciocalteu reagent. Methods Enzymol. 1998;299(1974):152‐178.

[45] Castro‐Alves VC , Cordenunsi BR . Total soluble phenolic compounds quantification is not as simple as it seems. Food Anal Methods. 2015;8(4):873‐884.

[46] Leyton A , Pezoa‐Conte R , Barriga A , et al. Identification and efficient extraction method of phlorotannins from the brown seaweed *Macrocystis pyrifera* using an orthogonal experimental design. Algal Res. 2016;16:201‐208.

[47] Zenthoefer M , Geisen U , Hofmann‐Peiker K , et al. Isolation of polyphenols with anticancer activity from the Baltic Sea brown seaweed *Fucus vesiculosus* using bioassay‐guided fractionation. J Appl Phycol. 2017;29(4):2021‐2037.

[48] Ragan MA , Glombitzka KW . Phlorotannins, brown algal polyphenols. Prog Phycol Res. 1986;4:129‐241.

[49] Glombitza KW , Schmidt A . Trihydroxyphlorethols from the brown alga *Carpophyllum angustifolium* . Phytochemistry. 1999;51(8):1095‐1100. 10.1021/np990076c 10514304

[50] Glombitza KW , Schmidt A . Nonhalogenated and halogenated phlorotannins from the brown alga *Carpophyllum angustifolium* . J Nat Prod. 1999;62(9):1238‐1240. 10514304 10.1021/np990076c

[51] Parys S , Kehraus S , Krick A , et al. *In vitro* chemopreventive potential of fucophlorethols from the brown alga *Fucus vesiculosus* L. by anti‐oxidant activity and inhibition of selected cytochrome P450 enzymes. Phytochemistry. 2010;71(2–3):221‐229. 19954804 10.1016/j.phytochem.2009.10.020

[52] Pantelidis GE , Vasilakakis M , Manganaris GA , Diamantidis G . Antioxidant capacity, phenol, anthocyanin and ascorbic acid contents in raspberries, blackberries, red currants, gooseberries and cornelian cherries. Food Chem. 2007;102(3):777‐783.

[53] Paiva L , Lima E , Neto A , Baptista J . Seasonal variability of the biochemical composition and antioxidant properties of *Fucus spiralis* at two Azorean Islands. Mar Drugs. 2018;16(8):248. 10.3390/md16080248 PMC6117708 30049966

[54] Jégou C , Kervarec N , Cérantola S , Bihannic I , Stiger‐Pouvreau V . NMR use to quantify phlorotannins: the case of *Cystoseira tamariscifolia*, a phloroglucinol‐producing brown macroalga in Brittany (France). Talanta. 2015;135:1‐6. 10.1016/j.talanta.2014.11.059 25640118

[55] Jégou C , Culioli G , Kervarec N , Simon G , Stiger‐Pouvreau V . LC/ESI‐MSn and 1H HR‐MAS NMR analytical methods as useful taxonomical tools within the genus Cystoseira C. Agardh (Fucales; Phaeophyceae). Talanta. 2010;83(2):613‐622. 10.1016/j.talanta.2010.10.003 21111182

[56] Keusgen M , Glombitza KW . Phlorethols, fuhalols and their derivatives from the brown alga *Sargassum spinuligerum* . Phytochemistry. 1995;38(4):975‐985.

[57] Glombitza KW , Keusgen M . Fuhalols and deshydroxyfuhalols from the brown alga *Sargassum spinuligerum* . Phytochemistry. 1995;38(4):987‐995.

[58] Grosse‐Damhues J , Glombitza KW . Isofuhalols, a type of phlorotannin from the brown alga *Chorda filum* . Tetrahedron Lett. 1984;23(11):2639‐2642.

[59] Cérantola S , Breton F , Gall EA , Deslandes E . Co‐occurrence and antioxidant activities of fucol and fucophlorethol classes of polymeric phenols in *Fucus spiralis* . Bot Mar. 2006;49(4):347‐351.

[60] Koch M , Glombitza KW , Rösener HU . Polyhydroxyphenyl ethers from *Bifurcaria bifurcata* . Phytochemistry. 1981;20(6):1373‐1379.

[61] Agregán R , Munekata PES , Franco D , Dominguez R , Carballo J , Lorenzo JM . Phenolic compounds from three brown seaweed species using LC‐DAD‐ESI‐MS/MS. Food Res Int. 2017;99(Pt 3):979‐985. 10.1016/j.foodres.2017.03.043 28865624

[62] Heffernan N , Brunton NP , FitzGerald RJ , Smyth TJ . Profiling of the molecular weight and structural isomer abundance of macroalgae‐derived phlorotannins. Mar Drugs. 2015;13(1):509‐528. 10.3390/md13010509 25603345 PMC4306949

[63] Montero L , Herrero M , Ibáñez E , Cifuentes A . Separation and characterisation of phlorotannins from brown algae *Cystoseira abies‐marina* by comprehensive two‐dimensional liquid chromatography. Electrophoresis. 2014;35(11):1644‐1651. 24723338 10.1002/elps.201400133

[64] Li Y , Fu X , Duan D , Liu X , Xu J , Gao X . Extraction and identification of phlorotannins from the brown alga, *Sargassum fusiforme* (Harvey) Setchell. Mar Drugs. 2017;15(2):1‐15. 10.3390/md15020049 PMC5334629 28230766

[65] Tierney MS , Soler‐Vila A , Rai DK , Croft AK , Brunton NP , Smyth TJ . UPLC‐MS profiling of low molecular weight phlorotannin polymers in *Ascophyllum nodosum*, *Pelvetia canaliculata* and *Fucus spiralis* . Metabolomics. 2014;10(3):524‐535.

[66] Novakova L , Matysova L , Solich P . Advantages of application of UPLC in pharmaceutical analysis. Talanta. 2006;68(3):908‐918. 10.1016/j.talanta.2005.06.035 18970409

[67] Audibert L , Fauchon M , Blanc N . Phenolic compounds in the brown seaweed *Ascophyllum nodosum*: distribution and radical‐scavenging activities. Phytochem Anal. 2010;21(5):399‐405. 20333652 10.1002/pca.1210

[68] Corona G , Coman MM , Guo Y , et al. Effect of simulated gastrointestinal digestion and fermentation on polyphenolic content and bioactivity of brown seaweed phlorotannin‐rich extracts. Mol Nutr Food Res. 2017;61(11):1‐10. 10.1002/mnfr.201700223 28718977

[69] Montero L , Sánchez‐Camargo AP , García‐Cañas V , et al. Anti‐proliferative activity and chemical characterization by comprehensive two‐dimensional liquid chromatography coupled to mass spectrometry of phlorotannins from the brown macroalga *Sargassum muticum* collected on North‐Atlantic coasts. J Chromatogr A. 2016;1428:115‐125. 26210109 10.1016/j.chroma.2015.07.053

[70] Edwards M , Watson L . Cultivating *Laminaria digitata* . Irish Fish Board. 2011;26(26):1‐71.

[71] Roy MC , Anguenot R , Fillion C , Beaulieu M , Bérubé J , Richard D . Effect of a commercially‐available algal phlorotannins extract on digestive enzymes and carbohydrate absorption *in vivo* . Food Res Int. 2011;44(9):3026‐3029.

[72] Heffernan N , Smyth TJ , Soler‐Villa A , Fitzgerald RJ , Brunton NP . Phenolic content and antioxidant activity of fractions obtained from selected Irish macroalgae species (*Laminaria digitata*, *Fucus serratus*, *Gracilaria gracilis* and *Codium fragile*). J Appl Phycol. 2014;27(1):519‐530.

[73] Wang T , Jónsdóttir R , Ólafsdóttir G . Total phenolic compounds, radical scavenging and metal chelation of extracts from Icelandic seaweeds. Food Chem. 2009;116(1):240‐248.

[74] Agregán R , Munekata PE , Domínguez R , Carballo J , Franco D , Lorenzo JM . Proximate composition, phenolic content and *in vitro* antioxidant activity of aqueous extracts of the seaweeds *Ascophyllum nodosum*, *Bifurcaria bifurcata* and *Fucus vesiculosus*. Effect of addition of the extracts on the oxidative stability of canola oil under accelerated storage conditions. Food Res Int. 2017;99:986‐994. 28865625 10.1016/j.foodres.2016.11.009

[75] Zhang Q , Zhang J , Shen J , Silva A , Dennis DA , Barrow CJ . A simple 96‐well microplate method for estimation of total polyphenol content in seaweeds. J Appl Phycol. 2006;18(3–5):445‐450.

[76] Stiger‐Pouvreau V , Jégou C , Cérantola S , Guérard F , Le LK . Phlorotannins in sargassaceae species from brittany (France). Interesting molecules for ecophysiological and valorisation purposes. Adv Bot Res. 2014;71:379‐411.

